# Novel Perspectives on p53 Function in Neural Stem Cells and Brain Tumors

**DOI:** 10.1155/2011/852970

**Published:** 2010-12-15

**Authors:** Sanna-Maria Hede, Inga Nazarenko, Monica Nistér, Mikael S. Lindström

**Affiliations:** Department of Oncology-Pathology, Karolinska Institute, CCK R8:05, SE-17176 Stockholm, Sweden

## Abstract

Malignant glioma is the most common brain tumor in adults and is associated with a very poor prognosis. Mutations in the p53 tumor suppressor gene are frequently detected in gliomas. p53 is well-known for its ability to induce cell cycle arrest, apoptosis, senescence, or differentiation following cellular stress. That the guardian of the genome also controls stem cell self-renewal and suppresses pluripotency adds a novel level of complexity to p53. Exactly how p53 works in order to prevent malignant transformation of cells in the central nervous system remains unclear, and despite being one of the most studied proteins, there is a need to acquire further knowledge about p53 in neural stem cells. Importantly, the characterization of glioma cells with stem-like properties, also known as brain tumor stem cells, has opened up for the development of novel targeted therapies. Here, we give an overview of what is currently known about p53 in brain tumors and neural stem cells. Specifically, we review the literature regarding transformation of adult neural stem cells and, we discuss how the loss of p53 and deregulation of growth factor signaling pathways, such as increased PDGF signaling, lead to brain tumor development. Reactivation of p53 in brain tumor stem cell populations in combination with current treatments for glioma should be further explored and may become a viable future therapeutic approach.

## 1. Introduction

The most frequent form of brain tumor in adults is glioma [1]. Gliomas are classified as astrocytomas, oligodendrogliomas, oligoastrocytomas, and ependymomas [2]. Astrocytoma is the most common subclass of glioma and is graded on a WHO scale of I to IV, whereas oligodendrogliomas and oligoastrocytomas are usually classified as grade II or grade III [3]. Grade IV astrocytic tumor, commonly known as glioblastoma (GB), is the deadliest form of brain tumor that despite multimodal therapy only shows a median survival of 12–15 months [4]. Recent transcriptome and genome profiling of brain tumors in combination with advances in stem cell biology has led to an improved understanding of the molecular pathology of this disease and revealed novel targets for therapy [5]. 

The p53 tumor suppressor gene is frequently mutated or deleted in human tumors and is often found mutated or lost early in glioma formation [6, 7]. p53 can trigger diverse cellular programs such as cell cycle arrest, apoptosis, differentiation, DNA repair, autophagy, and senescence [8]. One prevailing hypothesis is that GB could arise and recur because of malignant transformation of neural stem cells residing in protected niche areas [9]. Recently, novel functions of p53 in stem cells have been characterized including suppression of pluripotency and inhibition of stem cell self-renewal [10]. Despite being one of the most extensively studied proteins, there is still a need to acquire further knowledge and insight into p53 function in stem cells including neural stem cells. What function of p53 is the most important one to inactivate for brain tumor initiation and progression? Could it be the ability of p53 to restrain self-renewal and to promote differentiation, or is it the pro-apoptotic and cell cycle regulating activity? Here we discuss the role of p53 in gliomagenesis and the significance of p53 in relation to brain tumor stem cells. We review the literature regarding the neoplastic potential of neural stem cells, and we describe how the loss of p53 in parallel with deregulation of growth factor signaling pathways promotes brain tumor development. Finally, we discuss how the reactivation of p53 in brain tumor stem cell populations could become one viable approach to suppress proliferation and induce differentiation and apoptosis of these cells.

## 2. Glioma Genetics and Glioma Cell of Origin

### 2.1. p53 Pathway Inactivation in Glioma

Gliomas often display mutations in the ARF-MDM2-p53 and p16INK4a-CDK4-RB tumor suppressor pathways resulting in increased genomic instability, loss of G_1_ cell cycle checkpoint control, and evasion of apoptosis [2, 11]. Deregulation of the PI3K/AKT/mTOR signaling pathway and hyperactivation of receptor-tyrosine kinases (e.g., PDGFR*α* and EGFR) are frequently observed in gliomas [2, 11]. GBs can be classified as primary or secondary but are morphologically similar [1]. A primary GB arises with no signs of previous lower-grade tumor and often displays loss of the *INK4A/ARF* tumor suppressor gene locus, *PTEN* mutation, and *EGFR* amplification and/or mutation [1]. Secondary GBs show a previous history of progression from a lower-grade tumor and *TP53* mutations are frequent [2]. Recently, transcriptome and genome profiling of GBs has revealed additional genetic differences, and new subclasses of GB have been defined [12–14].

*TP53* mutations occur early in glioma progression, and grade II astrocytomas commonly display *TP53* mutations or loss of heterozygosity on chromosome 17p where *TP53 *resides [15, 16]. *TP53* mutations are infrequent in medulloblastomas, pilocytic grade I astrocytomas, and ependymomas [7]. The p53 tumor suppressor restricts cell growth and proliferation following stress and is known as the guardian of the genome [17]. p53 has pleiotropic anticancer functions and plays a role in senescence, apoptosis, differentiation, autophagy, metabolism, and angiogenesis [18]. These diverse cellular effects can be attributed to the regulation of hundreds of different genes directly by p53 [8, 19]. The transcriptional function of p53 is stimulated through increased levels of the protein coupled to conformational changes triggered by different posttranslational modifications or p53- binding proteins [20]. While approximately half of all human tumors contain a mutation or deletion of *TP53*, the rest of the tumors often have inactivation of p53 through other mechanisms including viral infection, loss of *ARF*, or overexpression of MDM2 [21]. The MDM4 and MDM2 proteins suppress p53's transcriptional activity and target p53 for proteasomal degradation, respectively [22]. It is therefore not a surprise to learn that MDM2 gene amplifications are present in 10% of primary GBs and that amplifications of *MDM4* are found in about 4% of GBs [23, 24]. Gliomas are also seen in the Li-Fraumeni syndrome, a familial cancer predisposing syndrome characterized by germ line *TP53* mutations [25, 26]. It has been a widely held notion that somatic *TP53 *mutations are common in low-grade gliomas and secondary GBs but more uncommon in primary GB. However recent studies, which also included additional sequencing of *TP53,* revealed that mutations are prevalent in primary GBs as well [13, 27]. Another common and critical tumor suppressor gene alteration in GB is loss of function of PTEN that occurs in both primary and secondary GB [28]. 

Accumulated experimental and clinical evidence suggests that the loss of p53 function is a key initial event in glioma development in combination with other genetic and epigenetic alterations [6, 7, 26, 29–34]. Numerous studies have also been carried out in order to investigate the effects of p53 overexpression in glioma cells. Evidently, p53 can block cell cycle progression and induce morphological changes resembling differentiation in glioma cell lines [35–37]. Given these findings, therapeutic targeting of the p53 pathway still seems highly interesting in glioma.

### 2.2. Neural Stem Cells

Tissue stem cells are considered to be rare cells within organs with the ability to self-renew and to give rise to all types of cells within the said organ [38]. Examples of tissue stem cells include hematopoietic stem cells, neural stem cells, and mammary gland stem cells [38]. Embryonic stem cells on the other hand are isolated from the inner cell mass of blastocysts, are pluripotent, and can give rise to all cell types of the body [39]. Neural stem cells are the self-renewing cells that generate the main cells of the central nervous system (astrocytes, neurons, and oligodendrocytes) [40]. New neurons are thought to be born throughout adulthood in predominantly two regions of the mouse brain [41]. These are the subventricular zone of the lateral ventricle wall, from where new neuronal progenitor cells migrate to the olfactory bulb via the rostral migratory stream [42, 43], and the subgranular zone of hippocampus [44]. Reynolds and Weiss were the first to isolate neural progenitor and stem cells from adult mouse brain [45]. Within the subventricular zone, cells can be classified as type B neural stem cells and type C transit-amplifying cells that give rise to neuroblasts (type A) [46]. Neural stem cells are often studied *in vitro* using a method referred to as the neurosphere assay developed by Reynolds and Weiss [45], see also [47] for an update. Neural stem cells have the properties of self-renewal, clonogenic capacity, and ability to engraft, migrate, and give rise to differentiated progeny [41, 46, 48].

### 2.3. Glioma Cell of Origin and the Cancer Stem Cell Hypothesis

Several common tumor forms, including brain tumors, have been shown to harbor a fraction of cells with stem-like features referred to as cancer stem cells [49]. The cancer stem cells are considered to be a relatively small population of cells that are capable of self-renewal, and the progeny of which can undergo differentiation to generate the phenotypic heterogeneity observed in solid tumors [50, 51]. Cancer stem cell populations have been found in many malignancies including those from breast [52], brain [53], pancreas [54], colon [55], and the hematopoietic system (acute myelogenic leukemia) [56]. Cancer stem cells show malicious behavior including prolonged exit from the cell cycle (quiescence), resistance to chemotherapeutic agents, efficient DNA repair, and resistance to apoptosis [57–60]. 

Using the approach of Reynolds and Weiss [45], several groups reported on the growth of adult and pediatric glioma cells cultured and propagated in the form of neurospheres, also known in this context as “gliospheres” [61–65]. The cells capable of self-renewal and of forming new gliospheres and with the ability to initiate the formation of a new tumor in nude mice are known as the brain tumor stem cells. These cells can also be referred to as brain tumor initiating cells or glioma stem cells. It was shown that gliospheres have an increased growth potential and features of aberrant differentiation when directly compared to normal neurospheres from the adult brain [66]. Cell sorting is based on expression of the cell surface marker CD133 selected for glioma cells with stem-like features [53], and CD133-positive brain tumor cells are relatively resistant to radiation when compared to CD133-negative cells [57]. The clinical value of CD133-expression in tumors remains unclear, but some groups have reported that the increased expression of CD133 is associated with a poor prognosis [67, 68]. However, recent studies indicate that also CD133-negative brain tumor cells can initiate tumor development and act as brain tumor stem cells [69].

It is at present discussed whether cancer stem cells represent a minority of the tumor cells [70]. If most cells within the tumor are endowed with stem cell properties, why should we focus our energy on targeting a specific sub-population of cells? Debated is also whether cancer stem cells originate from normal stem cells or from differentiated cells that have acquired the ability to self-renew [50], and this discussion is especially dynamic within the brain tumor research community [5, 71, 72]. It still remains unclear if gliomas (in general) originate from multipotent neural stem or progenitor cells, restricted neural progenitors, or mature glia cells that have undergone the process of de-differentiation [71].

## 3. Novel Functions of p53 in Neural Stem Cells

A number of recent studies show that p53 has a critical function in neural [73], mammary [74], hematopoietic [75, 76] and embryonic stem cells [77] by regulating self-renewal, symmetric division, quiescence, survival, and proliferation. Two main functions of p53 can be distinguished in relation to stem cell behavior. These can be described as the abilities of p53 to induce differentiation and to suppress de-differentiation and evidence in the literature supports a role for p53 in both of these processes [10, 78–81]. 

In the mouse brain, p53 is critical for induction of apoptosis in neural progenitors and postmitotic neurons during development of the central nervous system [33], and a subset of *Trp53* knockout mice develop exencephaly [82, 83]. Furthermore, neuronal cells are sensitive to p53-dependent apoptosis following irradiation, exposure to chemotherapeutic agents, and ischemia [84]. p53 regulates cell cycle progression and apoptosis but it can also directly modulate the transcription of genes that are specifically required for neuronal differentiation [81, 84]. The role of p53 in apoptosis of neuronal cells is relatively well understood [85, 86], but less is known about p53 function in astrocytes, oligodendrocytes and their precursors. Oligodendrocyte precursors cultured *in vitro* can undergo p53-dependent differentiation although the cells appear to have a low basal level of p53 expression [87]. Both astrocytes and oligodendrocytes can undergo apoptosis following infection with an adenovirus expressing p53 [88]. Although speculative, cells of the glial lineage may be more prone towards p53-induced differentiation and senescence following stress than neuronal cells. 

What is the function of p53 in the neural stem cells? The enhanced proliferative capacity of neural precursors from *Trp53* knockout mice was described in the early 90s [89]. Later, it was found that p53 is expressed at higher levels in cells in the neural stem cell niche than in other regions of the adult mouse brain [73]. Cells in the brain's lateral ventricle stem cell niche displayed an increased proliferation rate in *Trp53* knockout mice compared to wild-type [73]. It was also found that a p53 deficiency in neurospheres resulted in increased self-renewal capacity, increased cell proliferation and a reduction in apoptosis [73]. Analysis of the stem cell transcriptome from wild type and *Tp53* knockout mice identified several genes that were down-regulated in p53-null neurospheres, importantly p21 and p27, established negative regulators of cell proliferation [73]. In a related study, Gil-Perotin et al., found a p53-dependent effect on differentiationthe and they could determine that loss of p53 increased the number of Tuj1^+^ neuroblasts in the subventricular zone *in vivo * [31]. Regions with mild to moderate hyperplasia, resembling “microtumors” were also apparent in some *Trp53* knockout mice [31]. The increase in cell number was apparently not due to loss of apoptosis, as it could be compensated for, and it was argued that this is the reason for why there are no tumors in *Trp53* knockout mice [31]. Also by using olfactory bulb neural stem and progenitor cells cultured as neurospheres it was found, in agreement with the previous studies, that loss of p53 can promote neurosphere formation and stem cell self-renewal [90]. Furthermore, loss of p53 facilitated differentiation of progenitor cells into Tuj1-positive neurons, with a corresponding moderate decrease in mature astrocytes [90]. In summary, a number of studies show that the loss of p53 provides an advantage to neural stem cells and/or early progenitor cells [31, 73, 90]. However, the loss of p53 alone does not cause brain tumors within the relatively short life span of *Trp53* knockout mice [91]. 

p53-mediated control of stem cell functions has also been studied in other tissues. For example, p53 has a critical role in regulating hematopoietic stem cell quiescence [75, 76]. Transcriptome analysis identified *Gfi-1* and *Necdin* as p53 target genes involved in regulating quiescence [76]. In mammary gland stem cells, p53 controls polarity of mammary epithelial stem cell divisions [74]. In bone formation, the loss of p53 not only accelerates early osteogenesis from mesenchymal stem cells but actually prevents terminal differentiation to a mature osteocytic phenotype [92]. These studies taken together further strengthen the notion that p53 controls stem cell self-renewal and differentiation, but that it may not only do so in a cell-type-specific manner.

## 4. Brain Tumor Stem Cells and p53

### 4.1. Inactivation of p53 in Neural Stem Cells

Perhaps the suppression of incipient cancer stem cells is one activity by which p53 can inhibit tumor growth [8], but what are the mechanistic links between p53 and the emergence of cancer stem cells, if any? p53 was found to repress the cancer stem cell marker gene CD44 in an experimental breast tumor model [93]. Overexpression of CD44 on the other hand blocked p53-dependent apoptosis, leading to expansion of tumor-initiating cells [93]. It would be of interest to determine if similar mechanisms are involved also in brain tumor development, but exactly how the loss of p53 function leads to transformation of normal cells in the central nervous system remains unclear. Development of a brain tumor may begin with a mutation in the p53 gene which makes neural stem cells proliferate faster and perhaps also migrate out of the niche like their specialized progenies [9]. Wang and coworkers carried out a series of experiments using mice engineered to have an internal deletion mutation in *Tp53,* Δ exon 5-6, specifically in neural stem and progenitor cells [94]. They found that a majority of mice developed malignant brain tumors and that the same mutant p53 was detected in the tumor cells but not in normal cells. Mutant p53 protein was detectable in a minority of proliferative neural stem cells two months after birth. It was argued that it is presumably the mutant-p53-expressing cell population with features of transit-amplifying cells that drives tumor initiation [94]. The hypothesis that stem cells residing in the subventricular zone can give rise to gliomas is also supported by other studies [9, 29, 95, 96]. An interesting point of view is that tissue stem cells remain undifferentiated due to environmental cues in their particular niche, and the stem cells differentiate when they leave that niche, or no longer receive proper signals from the niche [97]. 

GB was initially considered to be a monoclonal tumor and the patterns of clonal expansion of cells with mutant p53 supported this notion [34]. However, given the heterogeneity of GB taken together with the recent findings that there are coexisting populations of cells with different p53 status within the same tumor, we must also consider polyclonal events [98]. GB is composed of several types of cells, and some phenotypes or clones may be better suited for the specific environment but they could still coexist with other sub-optimal lines of tumor cells [71]. One of these sub-optimal lines could however following a novel and different type of stress adapt and instead become the most successful line, and this type of event could contribute to treatment resistance of GB [5, 71]. Phenotypically different subpopulations of cells may even benefit from each other and thus remain coexisting in the tumor [99]. As with regard to p53, perhaps the majority of tumor cells benefit from having mutant p53 or no p53 at all, but may some tumor cells thrive when carrying wild-type p53?

### 4.2. Stem Cell Signatures in Brain Tumor Cells

Pluripotent stem cells can be generated from normal fibroblast cultures, and in principle four key pluripotency genes essential for the production of pluripotent stem cells were defined, namely: Oct-3/4, Sox2, c-Myc, and Klf4 [100]. Of interest is also the recently established function of the p53 pathway in suppressing reprogramming of normal cells to induced pluripotent stem cells (iPSCs) [101–104]. Silencing of p19ARF, an upstream regulator of p53, facilitates reprogramming as well [105]. It remains unclear how p53 blocks reprogramming of cells to iPSCs, but one possibility is that it could be related to the higher sensitivity of iPSCs to stress than the more differentiated cells from which they were initially derived [80]. The process of creating iPSCs resembles the creation of tumor cells by specific factors and highlights the similarity between iPSCs and cancer stem cells [79]. GBs frequently overexpress genes typical of neural stem cells including Sox2 [106], Myc [27] and Oct4 [107]. A hallmark of some poorly differentiated tumors, including GB, is a stem cell signature [108]. The malignant progression of glioma may be associated with the emergence of such a signature [109]. Therefore, targeting pluripotency-associated molecules such as Myc and Sox2, combined with reactivation of p53, specifically in brain tumor stem cell populations could become one approach to effectively reduce tumor growth. In fact, c-Myc is required for brain tumor stem cell growth [110], and in normal neural stem cells, loss of c-Myc on its own attenuates self-renewal and induces differentiation towards the glial lineage [111]. 

In this context it should also be mentioned that p53 plays a specific role in the DNA-damage response of embryonic stem cell [77]. Embryonic stem cells lack a distinct G_1_/S cell cycle checkpoint [112], but new evidence shows that p53 in response to DNA damage acts to induce differentiation and to suppress expression of the pluripotency factor Nanog [77]. Whether a similar mechanism is involved in the neural stem cell stress response, remains of interest to determine.

### 4.3. Other Regulators of p53 in Brain Tumor Stem Cells

We must also take into account the function and expression of proteins and microRNAs that regulate p53. Olig2 is a central-nervous-system-restricted transcription factor highly expressed in brain tumor stem cells and required for neural progenitor cell proliferation [113]. Olig2 directly suppresses p21, a downstream key target of p53, and Olig2 is therefore presumably an important antagonist of the p53 pathway during glioma development [113]. Interestingly, loss of p21 increases the proliferative capacity of neural stem cells [114]. Another important regulator of p53 is Gli1, a downstream mediator of Hedgehog signaling. Gli1 can repress p53 activity and Gli1 promotes an increase in neural stem and progenitor cell pools [115]. However, p53 in turn can suppress Gli1 function and proper Gli1 subcellular localization [116]. Interestingly, Gli1 function also depends on Nanog [116]. These studies revealed novel intricate signaling networks in stem cells and how they are connected to p53 [117]. Recently, Bcl2L12 (Bcl2-like 12) a protein found overexpressed in GB that prevents apoptosis, was shown to interact with and inhibit p53 [118]. As mentioned, loss of the tumor suppressor PTEN is a frequent event in brain tumors [28]. Interestingly, PTEN is critical in restricting neural stem cell self-renewal and proliferation similar to p53 [119, 120], and the combined loss of p53 and PTEN promotes a synergistic increase in neural stem cell self-renewal associated with elevated levels of c-Myc and rapid growth of gliomas *in vivo * [27, 121]. In turn, c-Myc promotes an even more malignant phenotype of the tumor [27]. Finally, regulation of the p53 pathway by microRNAs is likely to be of importance also in brain tumor development and brain tumor stem cells [122].

## 5. Interplay between p53 and Overactive PDGF Signaling

A number of recent studies using animal models have provided compelling evidence that gliomas can be induced from neural stem cells, provided combinations of several different tumor suppressors are deleted (e.g., *Pten, Nf1* and *Trp53)* [29, 94–96]. Loss of p53 in neural stem cells has in mouse models been proven as an important step in the initiation of gliomas [123]. Using a different approach, others have shown that persistent mitogen signaling (e.g., PDGF-B) can promote gliomagenesis also in lineage- restricted progenitor cells giving rise to oligodendroglioma-like tumors [124]. Therefore, development of gliomas may take divergent pathways and start in different cell types and locations [5]. Oligodendrocyte progenitor cells express PDGFR*α* and can be induced to proliferate when stimulated with PDGF [125]. The use of retrovirus or adenovirus vectors to introduce PDGF-B in newborn mice brains mostly results in Gfap−/Ng2+/Olig2+ tumors that by transcriptome analysis are similar to oligodendrogliomas [126–128]. This is true even if the virus is directed to neural precursors by the Nestin-tva or Gfap-tva systems [129, 130]. The oligodendroglioma-like features have been interpreted as due to PDGF's ability to modulate the balance between neuronal and glial cells generated from neural stem cells, in favor of oligodendrocyte progenitors [127].

In a recent report, transgenic mice were generated expressing PDGF-B in brain under control of the human GFAP promoter [32]. These mice were shown to be similar to wild type mice, but on a *Tp53-*null background they developed large GB-like brain tumors at a high frequency, in spite of the fact that *Tp53-*null mice do not otherwise develop brain tumors [32, 91]. The tumors were very heterogenous, displaying many different cell lineage markers, including stem cell markers. Early lesions displayed abundant Gfap-positive cells, although the larger tumors partly lost the expression of Gfap. The Gfap promoter is most active around birth and remains active in both astrocytes and neural stem cells of the adult brain; however; the mice developed brain tumors only in adult life, at 2–6 months of age [32]. Therefore, distinct possibilities of glioma cells of origin need to be considered including (1) adult neural stem cells that lose their differentiation capacity and (2) mature astrocytes that dedifferentiate due to the lack of p53. These PDGF-induced experimental gliomas are similar to human GBs in that the glial tumor cells express Pdgfr*α* whereas the vasculature expresses Pdgfr*β*  in pericytes [131]. The model was created to mimic human secondary GBs characterized by combined *PDGFRA* overexpression/amplification and *TP53* deletion/mutation, and results did prove that this combination is instrumental in generating GBs. A previous report described a significant association between *PDGFRA* expression, as analyzed at the mRNA level by *in situ* hybridization and LOH17p in human gliomas [132]. Recently, by the help of high-throughput genome and transcriptome analyses, human secondary GBs were shown to be similar to the proneural type of primary GBs and associated with *PDGFRA* amplification, *TP53* and *PTEN* deletion/mutation, *IDH1* mutation and also disturbances in the PI3K signaling pathway, and finally by the expression of oligodendrocyte markers [133].

Loss of function of p53, together with overactive growth factor signaling, contributes to glioma formation. In the transgenic mice expressing PDGF-B in astrocytes, tumors developed in homozygously deleted *Trp53* but not in heterozygous mice [32]. Furthermore, PDGF-B retrovirus-induced brain tumors developed at a higher frequency and with shorter latency when injections were performed in *Trp53-*null than in wild type mice, and in a *Trp53-*null background these tumors showed higher p-Akt and lower Pten levels [134]. Still, the mechanism of the combined PDGF-B/*Trp53 *null effect has not yet been clearly defined. One guess is that the *Trp53-*null status directly or indirectly allows for proliferation of Pdgfr*α*-positive precursors in the brain and/or for upregulation of *Pdgfra* expression at the promoter level. Cultured, otherwise normal *Trp53-*null brain cells show an increased survival and proliferation *in vitro,* coupled with upregulation and activation of Pdgf-receptors [134]. Other mechanisms need to be considered as well. PDGF signaling is known to expand the pool of glial progenitors generated from neural stem cells [135]. Given the fact that the lack of p53 may in addition result in an undifferentiated state of these cells, an increase in the number of glial precursors promoted by PDGF, possibly induced to migrate [136], but unable to differentiate further, may be all that it takes to create a lethal neoplasm in the mouse brain. We need to consider that the human brain has tighter control mechanisms than the mouse brain, but The Cancer Genome Atlas (TCGA) and other similar high-throughput screening projects provide us with excellent tools to identify additional molecules and mechanisms affected in human brain tumors [12].

## 6. Therapeutic Opportunities

Treatment of brain tumor patients is extremely challenging because the normal brain is highly susceptible to damage during therapy, the brain has a very limited capacity to repair itself, and several drugs cannot cross the blood-brainbarrier to act on tumors in the CNS [5]. Brain tumor cells are also highly infiltrative and can hide in apparently normal parts of the brain [137]. Paradoxically, nonmalignant neuronal cells are highly vulnerable to stress and respond with the induction of p53-dependent apoptosis [84], yet glioma-derived cells show resistance to apoptosis-inducing stimuli [138]. Standard treatment for GB is surgery, radiation therapy and concomitant and adjuvant treatment with the chemotherapeutic agent temozolomide [139]. Temozolomide is an alkylating agent found to have beneficial effects in the palliative treatment of GB [140]. Whereas glioma cell lines expressing mutant p53 are sensitized to temozolomide, the status of p53 does not seem to affect the response of brain tumor stem cells treated with this drug [141]. Despite the fact that the survival outlook for GB patients remains poor, recent years have seen progress towards longer survival, as summarized in an excellent review [4]. Several targeted therapies are currently in preclinical or clinical phase I–III trials and examples include small molecule or antibody inhibitors of receptor tyrosine kinases, angiogenesis regulators, histone deacetylases, heat shock proteins and mTOR [4, 142]. Early results from monotherapy trials have been rather disappointing, but a number of emerging drug candidates used in combination are expected to further prolong survival of brain tumor patients [4, 142]. 

What about specific targeting of brain tumor stem cells? As mentioned, brain tumor stem cell populations show resistance to drugs and toxic agents, display efficient DNA repair, and low tendency to undergo apoptotic cell death [72]. Screenings to find novel small molecules that specifically target cancer stem cell populations have been carried out. Salinomycin is a novel small molecule that targets breast tumor stem cells and selectively reduces the proportion of these cells relative to the effect of paclitaxel [143]. Development of similar drugs to be used in brain tumor therapy is therefore desired. Molecular targets in brain tumor stem cell populations could for instance be different components of the PTEN-PI3K-AKT-WNT signaling networks that drive cell growth [144]. Another approach could be to target brain tumor stem cells with small molecules that can induce differentiation, for example, histone deacetylase inhibitors [145]. Ribosomal DNA transcription (the RNA pol I machinery) is also an emerging target in cancer therapy [146]. Depleting cells of ribosomes by blocking production of ribosomal proteins was shown to induce p53-dependent inhibition of cell proliferation and morphological differentiation of glioma cells *in vitro* [147]. Selective inhibition of ribosome biogenesis in stem-like cell populations in brain tumors as another kind of differentiation therapy should be explored further. 

One prime candidate is of course p53 itself. Restoration of the p53 tumor suppressor function holds promise in cancer therapy [148, 149]. Tumors with dysfunctional or no p53 have been shown to undergo apoptosis or senescence *in vivo* upon functional restoration of p53 [150, 151]. When it comes to the tumor cells, we first need to distinguish cells with no p53 from cells with mutant p53, and cells retaining wild-type p53. Activation of endogenous wild-type p53 with small molecules, reactivation of mutant p53, or transfer of the p53 gene should therefore be considered [152]. Nutlin-3 is a compound that disrupts the binding between MDM2 and p53 leading to activation and accumulation of free p53 [153]. Interestingly, activation of endogenous wild type p53 with Nutlin-3 correlated with restoration of asymmetric breast cancer stem cell divisions resulting in tumor reduction [74]. This occurred in the absence of any major effects on the bulk of tumor cells [74]. In another study it was found that mutant p53 reactivation with the drug ellipticine when combined with 5-fluorouracil led to depletion of colon cancer stem cells *in vitro* [154]. Perhaps the reactivation of p53 specifically in brain tumor stem cells could induce permanent differentiation or apoptosis followed by tumor regression? Apoptosis is however not frequently seen upon retroviral expression or activation of endogenous p53 in glioma cell lines [36], but overexpression of p53 by adenovirus may sensitize glioma cells to apoptosis [155]. Indeed, adenoviral expression of p53 has been extensively tested in glioma [156].

There are some pitfalls when it comes to p53 reactivation that need to be discussed. For instance, maintaining wild type p53 could have a prosurvival effect on tumors that are not intrinsically prone to apoptosis [157]. In tumors resistant to cell death, p53 may favor DNA repair and differentiation over apoptosis or senescence [157]. For example, wild-type-p53-containing glioma cell lines are more resistant to cytotoxic agents than cell lines with mutant p53 [158]. We must also take into consideration that activation of p53 may lead to the emergence of treatment resistant brain tumor cells that express mutant p53 or that have completely lost p53. Moreover, persistent activation of p53 in nearby residing normal neural stem cells could have adverse negative sideeffects such as stem cell depletion and premature organ aging [58, 159]. Whereas activation of p53 traditionally has been viewed as the main avenue, the potential medical applications of inhibiting p53 should also be realized. In fact, inhibiting p53 protects normal cells from radiation-induced cell death and can improve recovery after ischemia in the central nervous system [160]. Unfortunately, inhibition of p53 activity in nontumorigenic cells could have a procarcinogenic effect, although encouraging results from studies in hematopoietic cells indicate that this might not be the case [161].

## 7. Concluding Remarks

There are a number of remaining unresolved issues with regard to the existence and phenotype of brain tumor stem cells and how similar they are to normal neural stem cells [5, 71]. The hypothesis that a normal neural stem or progenitor cell can evolve to become a brain tumor stem cell perhaps through a mutation in p53 is a very reasonable one and has got substantial experimental support [27, 29, 31, 94]. Several lines of evidence in the literature indicate that loss of p53 affects the properties of adult neural stem cells by providing a proliferative advantage [31, 73, 90]. Although loss of p53 on its own does not give rise to brain tumors in mice, it allows for rapid tumor development in the presence of persistent mitogen signaling, oncogene activation and subsequent mutational events [32]. However, we must also be aware that there are presumably different cellular origins for gliomas and that they could originate from various regions of the brain [5]. We are now faced with the intriguing situation in which p53 suppresses tumor development by restricting expansion of incipient brain tumor stem cells, but p53 also retains its conventional roles in controlling cell cycle progression and apoptosis following stress. Which one of these p53 activities is the most important one to circumvent during gliomagenesis, remains to be determined. We also do not know exactly through which mechanisms and pathways p53 controls neural stem cell self-renewal, but once identified, such mechanisms are putative novel targets for therapy. Treatment of brain tumor patients presents a unique challenge, and the selective targeting of brain tumor stem cell populations, regardless of whether they constitute a minor or major part of the whole tumors needs to be further explored, in order to become a clinical reality in the future. Combination therapies targeting both brain tumor stem cells and bulk brain tumor cells could turn out to be the most effective ones. It is of outermost importance to achieve a permanent eradication of brain tumor cells including brain tumor stem cells. However, the elimination of all tumor cells remains a very difficult task when considering the wide spectrum of tumor cell phenotypes, differences in p53 status, and divergent cellular responses to treatment.
